# A new species of *Polyneura* Westwood, 1842 from Yunnan, China (Hemiptera, Cicadidae, Cicadinae)

**DOI:** 10.3897/BDJ.10.e84554

**Published:** 2022-06-02

**Authors:** Cheng-Bin Wang, Peng-Yu Liu

**Affiliations:** 1 Engineering Research Center for Forest and Grassland Disaster Prevention and Reduction, Mianyang Normal University, 166 Mianxing West Road, Mianyang, China Engineering Research Center for Forest and Grassland Disaster Prevention and Reduction, Mianyang Normal University, 166 Mianxing West Road Mianyang China; 2 Fuzhou Chonglinyequ Cultural creativity Co., Ltd., Taijiang District, Fuzhou, China Fuzhou Chonglinyequ Cultural creativity Co., Ltd., Taijiang District Fuzhou China

**Keywords:** Cicada, Polyneurini, Polyneurina, taxonomy, morphology, new species, Oriental Region

## Abstract

**Background:**

The tribe Polyneurini Amyot & Audinet-Serville, 1843 (Hemiptera: Cicadidae: Cicadinae) is a small tribe which includes four genera and 18 species, distributing in eastern Palaearctic and Oriental Regions. Most of them, four genera and 16 species, are known from China.

**New information:**

A remarkable new species of cicada, *Polyneuraguoliangi* **sp. n.** (Hemiptera, Cicadidae, Cicadinae), is described and illustrated from Yunnan, southwest China. The diagnostic characters of the new species are compared with allied species or genera.

## Introduction

The tribe Polyneurini Amyot & Audinet-Serville, 1843 (Hemiptera, Cicadidae, Cicadinae) can be separated from related tribes by a combination of the following characters: rostrum extending beyond hind coxae; wings opaque or translucent; venation with numerous longitudinal veins; nodal line distinct or visible; male opercula large, slightly overlapping or adjacent to each other at middle, apex not exceeding middle of abdomen; uncus of pygofer swollen ventrally, not concealed with pygophore; uncus lobes largely separated from each other from base; female abdomen shorter than length from tip of head to cruciform elevation; ovipositor not extending beyond abdominal segment IX (Hayashi 1978, Chou et al. 1997). Boulard (2005) reviewed the history of Polyneurini and commented on some synonymies in the tribe. Later on, the subtribe Formotosenina was established to accommodate the genus *Formotosena* Kato, 1925 and the other three genera, *Polyneura* Westwood, 1842, *Graptopsaltria* Stål, 1866 and *Angamiana* Distant, 1890 were placed in the subtribe Polyneurina Myers, 1929 accordingly (Boulard 2008).

For the type genus *Polyneura* Westwood, 1842, six valid species were recorded before this study (Sanborn 2013). Westwood (1840) first recorded *P.ducalis* Westwood, 1840 from India. Almost a century and a half later, Chou and Yao (1986) described five species from south China: *P.cheni* Chou & Yao, 1986 from Sichuan, *P.xichangensis* Chou & Yao, 1986 from Sichuan, *P.parapuncta* Chou & Yao, 1986 from Guizhou, *P.tibetana* Chou & Yao, 1986 from Xizang and *P.laevigata* Chou & Yao, 1986 from Jiangxi.

In 2019, Mr. Liang Guo (Fuzhou, China) collected three species of Polyneurini at Xiaoheishan Nature Reserve (Yunnan, southwest China) from 9-15 September by using a net with an extension handle (11 m): *Angamianafloridula* Distant, 1904, *Polyneuracheni* Chou & Yao, 1986 and a remarkable unkown species of *Polyneura*. However, only one male of the unkown species was obtained. Herein, we describe it under the name of *Polyneuraguoliangi* **sp. n.** Important morphological characters of the new species are illustrated. Its unique morphological features are discussed by comparing with allied species or genera and a key to all species of *Polyneura* from China is provided.

## Materials and methods

The specimen was relaxed and softened in water at room temperature for 24 hours and then placed in distilled water for cleaning and dissection. To examine the male genitalia, the pygofer (containing aedeagus) together with sternite VIII were detached and treated with a 10% potassium hydroxide (KOH) solution at room temperature for 12 hours. They were then placed in distilled water to remove the remaining KOH and prevent any further bleaching. After examination, the body parts were mounted on a slide using Euparal Mounting Medium for future studies. Images were taken with a Canon macro photo lens MP-E 65 mm on a Canon 5DsR. Images of the same object at different focal planes were combined using Zerene Stacker 1.04 stacking software. Adobe Photoshop CS6 was used for post-processing. Morphological terminology follows Moulds (2005), Moulds (2012) and higher taxonomy follows Sanborn (2013).

The material examined for this study is deposited in the following institutional and private collections: **CLGF**: Collection of Liang Guo, Fuzhou, CHINA; **MYNU**: Invertebrate collection of Mianyang Normal University, Mianyang, CHINA.

Measurement criteria in millimetres (mm) are as follows: **body length**: length between the tip of head and the abdominal apex along the mid-line; **head length**: length between the tip of head and the anterior border of pronotum along the mid-line; **head width**: widest part of head (including eyes); **pronotal length**: length between the basal and apical borders of pronotum along the mid-line; **pronotal width**: widest part of pronotum (including pronotal collar); **mesonotal length**: length between the basal border of pronotum and apical border of cruciform elevation along the mid-line; **mesonotal width**: width at the base of mesonotum; **forewing length**: length between the base and the apex of forewing; **forewing width**: widest part of forewing; **abdominal length**: length between the apical border of cruciform elevation and the abdominal apex along the mid-line.

## Taxon treatments

### Polyneura guoliangi

Wang & Liu sp. n.

B245A11E-F87C-54DE-9CD0-E03CEECD135E

F6C6DFD3-A199-4603-B8DA-368ED892B4D6

#### Materials

- **Type status:** Holotype. **Occurrence:** recordedBy: Liang Guo; sex: male; **Taxon:** scientificName: *Polyneuraguoliangi* Wang & Liu; family: Cicadidae; genus: Polyneura; specificEpithet: *guoliangi*; **Location:** country: CHINA; stateProvince: Yunnan; verbatimLocality: Baoshan City, Tengchong, Xiaoheishan Nature Reserve [小黑山自然保护区]; verbatimElevation: 2150 m; **Event:** verbatimEventDate: 12.IX.2019; **Record Level:** institutionCode: MYNU

#### Description

**Figure 1.**
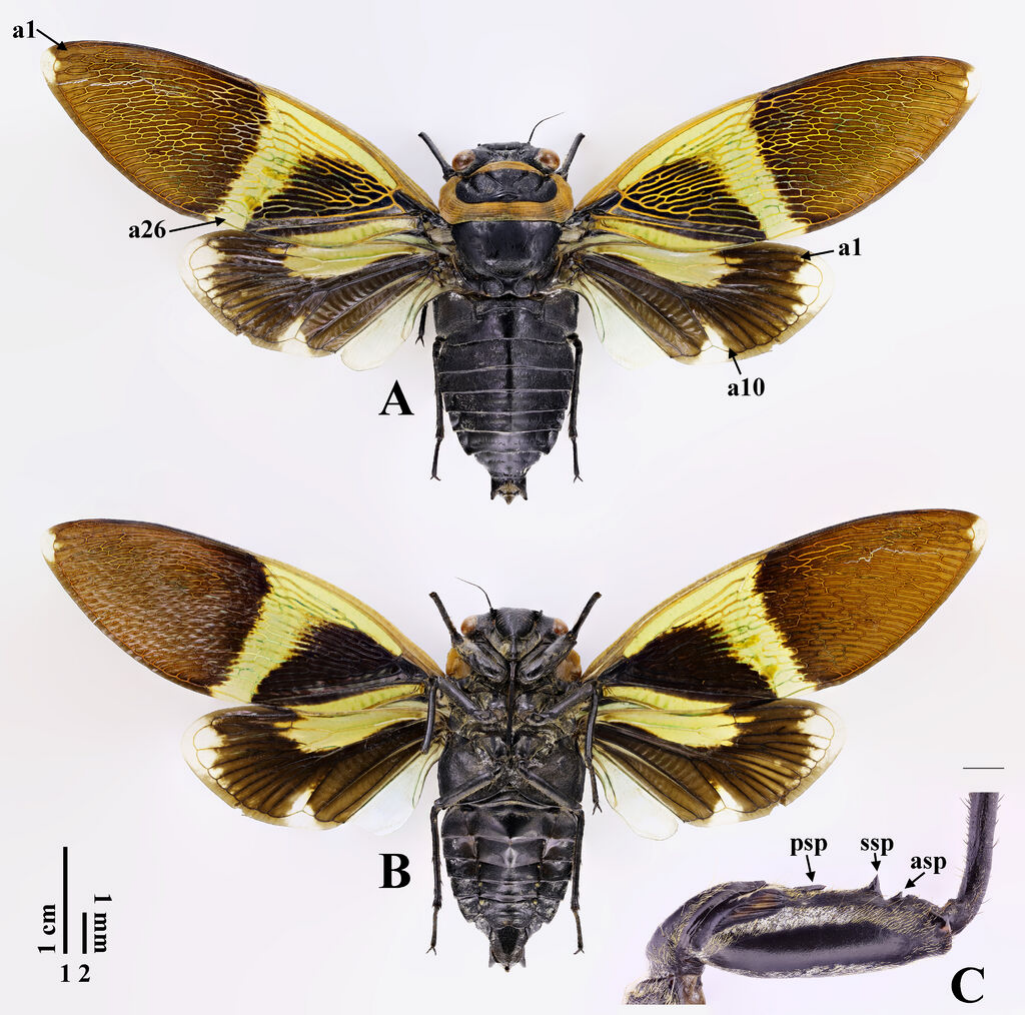
*Polyneuraguoliangi* **sp. n.**, holotype, male: **A** habitus, dorsal view; **B** habitus, ventral view; **C** fore femur, lateral view. Abbreviations: a1, 10, 26: apical cells 1, 10, 26; asp: subapical spine; psp: primary spine; ssp: secondary spine. Scale bar 1 for A and B; 2 for C.

**Male holotype** (Fig. 1A and B). Measurements (n = 1). Body 33.9 mm long. Length of different body parts (mm): head (1.7), pronotum (5.6), mesonotum (6.9), forewing (40.5), abdomen (19.9); width: head (10.2), pronotum (12.7), mesonotum (10.2), forewing (14.1).

Head mostly black, about 4/5 width of pronotum and as wide as mesonotal base. Compound eyes and ocelli brown. Distance between lateral ocellus and corresponding eye about 2.6 times as wide as distance between lateral ocelli. Postclypeus moderately swollen, with setigerous transverse grooves on each side. Anteclypeus and lorum densely covered with yellowish setae. Rostrum black, only brown at apex, extending to hind coxae.

Thorax. Pronotum black, 3.3 times as long as head and about 1.1 times as long as mesonotum excluding cruciform elevation, with transverse yellowish-brown fascia along anterior border. Pronotal collar yellowish-brown, wide, moderately ampliate laterally, lateral margins sinuate, but not dentate, hind corners widely rounded, suface transversely grooved. Mesonotum entirely black. Cruciform elevation black. Thoracic sternites black.

Legs entirely black. Fore femur (Fig. 1C) with wide, curved seta band in middle of lateral surface; primary spine thin, procumbent and pointed; secondary spine trianglular, erect and pointed; subapical spine subtrianglular, short and obtuse.

Wings generally opaque. Forewing with 26 apical cells; nodal line visible; yellowish in radial cell, wide cross band around nodal line and clavus; blackish before cross band and blackish to yellowish-brown after cross band; translucent at wing apex (including apical part of apical cell 2); costa vein and R+Sc vein yellowish-brown to brown. Hind-wing with 10 apical cells; yellowish in costal cell, radial cell and basal parts of apical cells 1–2 or 3 or 5; translucent at wing apex (including apical parts of apical cells 1–6), apical parts of apical cell 10 and cubital cell 1 (including marginal area after them) and anal lobe; other parts brown.

Abdomen entirely black, about 1.4 times as long as length from tip of head to cruciform elevation. Timbal cover large, scalloped, completely concealed timbal. Operculum short, wider than long, overlapping to each other at middle and with rounded apex just reaching posterior margin of abdominal sternite II. Abdominal sternite VII subtrapezoidal, widely emarginate at middle of posterior margin; sternite VIII oblong, narrowly emarginate at middle of posterior margin.

**Figure 2.**
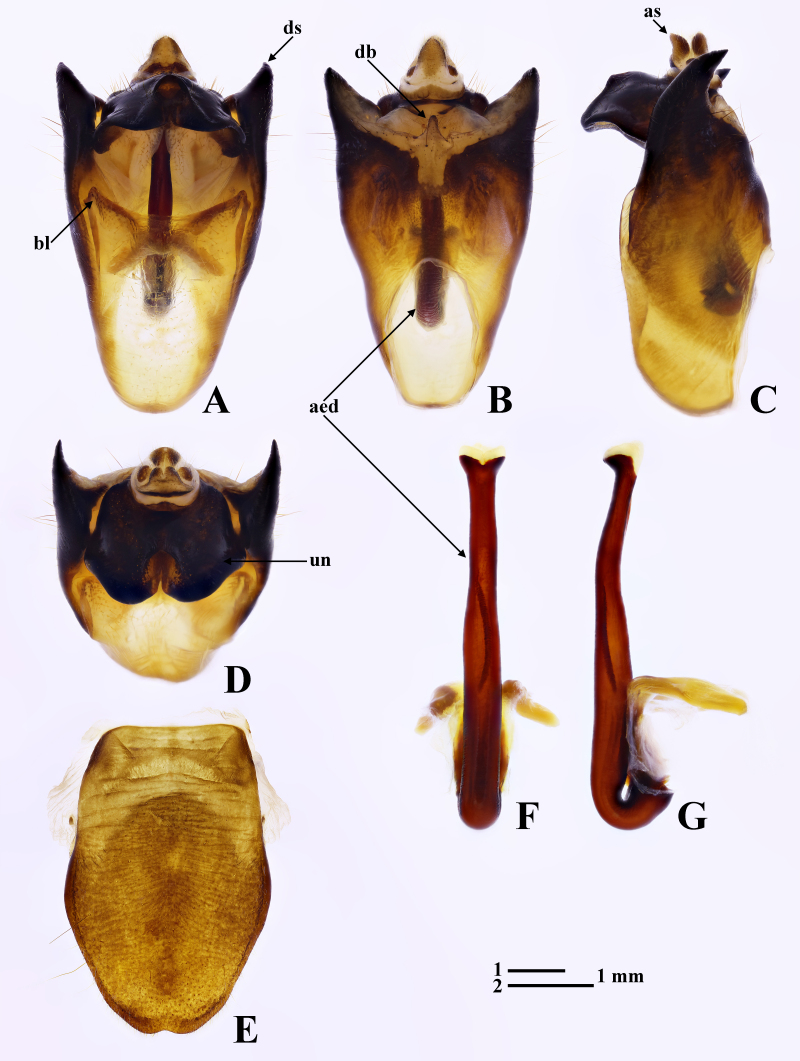
*Polyneuraguoliangi* **sp. n.**, holotype, male. **A** pygofer, ventral view; **B** pygofer, dorsal view; **C** pygofer, lateral view; **D** pygofer, apical view; **E** abdominal sternite VIII, ventral view; **F** aedeagus, ventral view; **G** aedeagus, lateral view. Abbreviations: aed: aedeagus; as: anal styles; bl: basal lobe of pygofer; db: dorsal beak; ds: distal shoulder; un: uncus. Scale bar 1 for A–E; 2 for F and G.

Male genitalia. Pygofer (Fig. 2A and B) subtrianglular, gradually narrowing towards base; anal styles (Fig. 2A–D) less sclerotised, densely covered with short setae; dorsal beak (Fig. 2B) less sclerotised, papillary; basal lobes (Fig. 2A) less developed, short; upper lobes absent; distal shoulders (Fig. 2A–B) strongly developed, large, noticeably divergent, with long hairs in lateral surfaces and sharply curving dorsally in lateral view (Fig. 2C); uncus (Fig. 2D) short and robust, wider than long, surface with hairs arranging in pair of oblique lines, rounded apices bifurcate from each other from half length and slightly deflexed at apex in lateral view (Fig. 2C). Aedeagus (Fig. 2F–G) long and slender, slightly bilobate at apex, with margins gently sinuous.

**Female**: Unkown.

#### Etymology

The new species is dedicated to the collector of the type specimen, Mr. Liang Guo (Fuzhou, China), an enthusiastic amateur entomologist. The name is a noun in the genitive case. The Chinese name “郭亮网翅蝉” is proposed for the Chinese common name of this new species.

#### Distribution

**Figure 3.**
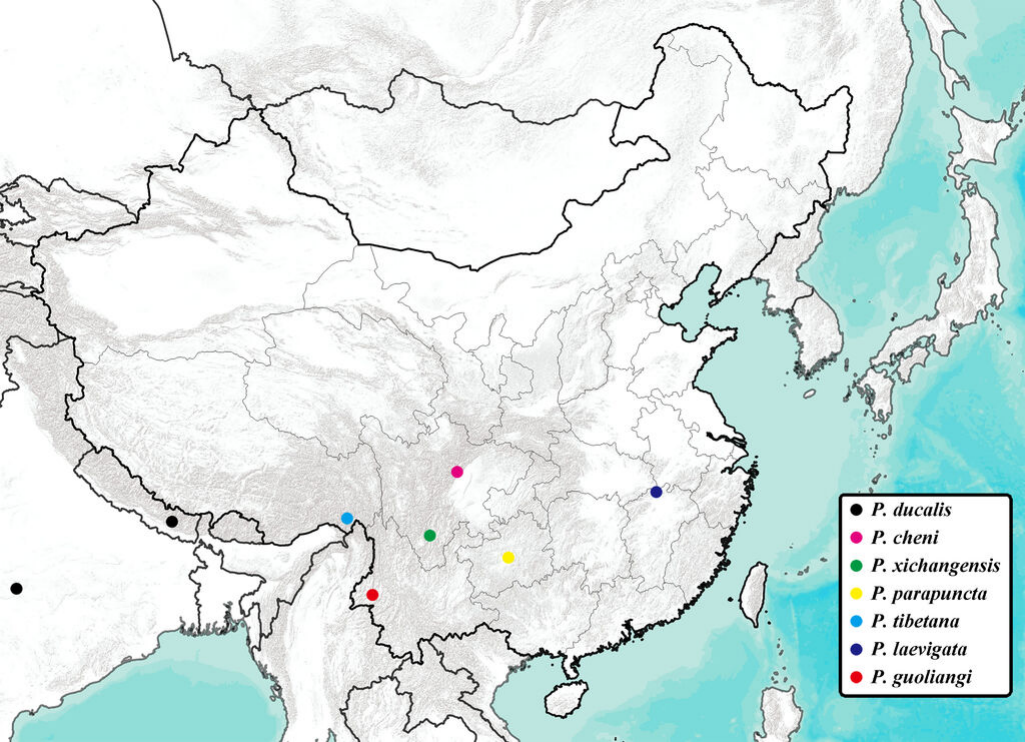
Map of type localities of *Polyneura* species. Note: Type localities of *P.ducalis* Westwood, 1840 and *P.parapuncta* Chou & Yao, 1986 are only known at the national level and provincial level, respectively, so they were roughly located at the centre of India and Guizhou (China).

China (Yunnan) (Fig. 3).

#### Taxon discussion

For comparison, the following material was studied: ***Formotosenamontivaga* (Distant, 1889). Material examined. THAILAND**: 1♂, Doi Saket, IX.2017, local people leg. (CLGF). ***Angamianafloridula* Distant, 1904. Material examined. CHINA**: 1♂2♀♀, Yunnan, Baoshan City, Tengchong, Xiaoheishan Nature Reserve [小黑山自然保护区], 2150 m, 9–15.IX.2019, Liang Guo leg. (CLGF). ***Graptopsaltriatienta* Karsch, 1894. Material examined. CHINA**: 1♂, Fujian, Nanping City, Wuyishan Nature Reserve [武夷山自然保护区], 21.VIII.2017, Liang Guo leg. (CLGF). ***Polyneuracheni* Chou & Yao, 1986. Material examined. CHINA**: 4♂♂6♀♀, Sichuan, Liangshan Prefecture, Mianning, VIII.2020, Wei Xie leg. (MYNU); 2♂♂2♀♀, Yunnan, Baoshan City, Tengchong, Xiaoheishan Nature Reserve [小黑山自然保护区], 2150 m, 9–15.IX.2019, Liang Guo leg. (CLGF).

It is doubtless that this new species belongs to Polyneurina, Polyneurini as indicated by Hayashi (1978), Chou et al. (1997). Most diagnostic characters of the new species are within the genus *Polyneura* with the peculiar synapomorphy of the wing venations (as an autapomorphy) and the shape of the male opercula. Except for its unique colour pattern on wings, it is also easy to distinguish the new species from other *Polyneura* congeners by the following characters: in *Polyneuraguoliangi* **sp. n.**: pygofer with distal shoulders strongly developed, large, noticeably divergent; uncus wider than long, with uncus lobes much wider and stout, divergent from half length. While in its congeners: pygofer with distal shoulders underdeveloped, small; uncus longer than wide, with uncus lobes relatively short and thin, divergent from basal part.

As the new species is really unique in morphological features, we would like to compare the differences between it and allied genera:

In *Graptopsaltria* Stål, 1866: forewing with eight apical cells; abdomen shorter than length from tip of head to cruciform elevation; uncus of pygofer longer than wide, with uncus lobes widely divergent from basal part, becoming closer to each other at apices. While in *Polyneuraguoliangi* **sp. n.**: forewing with 26 apical cells; abdomen longer than length from tip of head to cruciform elevation; uncus of pygofer wider than long, with uncus lobes much wider and stout, divergent from half length.

In *Angamiana* Distant, 1890: forewing with about 13 apical cells; male operculum obviously longer than wide; uncus of pygofer longer than wide, with uncus lobes much long and slender, divergent from basal part and distinctly deflexed at subapex. While in *Polyneuraguoliangi* **sp. n.**: forewing with 26 apical cells; male operculum wider than long; uncus of pygofer wider than long, with uncus lobes much wider and stout, divergent from half length and slightly deflexed at apex.

In *Formotosena* Kato, 1925: forewing with eight apical cells; male timbal cover incompletely concealed timbal which is partly exposed in dorsal view; male opercula nearly touching, but separated from each other; uncus of pygofer longer than wide, with uncus lobes relatively short and wide, divergent from basal part. While in *Polyneuraguoliangi* **sp. n.**: forewing with 26 apical cells; male timbal cover completely concealed tymbal; male opercula slightly overlapping to each other at middle; uncus of pygofer wider than long, with uncus lobes much wider and stout, divergent from half length.

#### Notes

**Figure 4.**
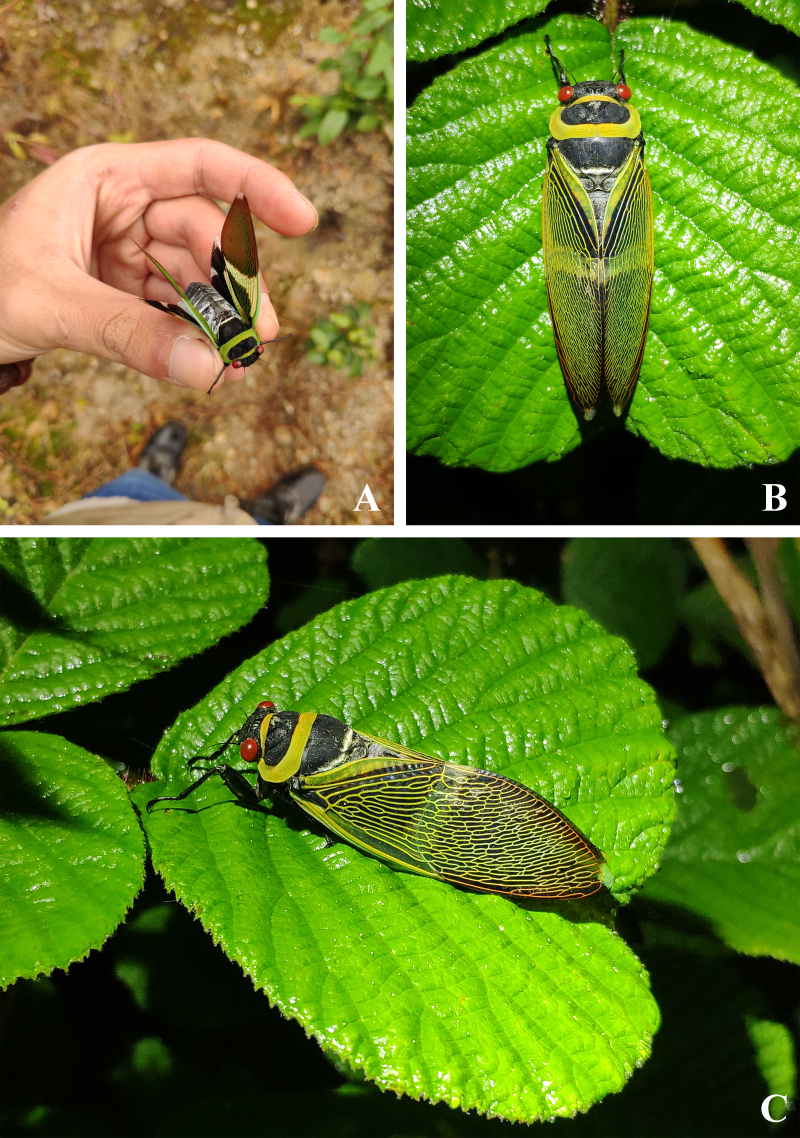
Living *Polyneuraguoliangi* **sp. n.** (holotype, male) at Xiaoheishan Nature Reserve (Yunnan, China). **A** caught by the collector Liang Guo; **B** dorsal view; **C** dorsolateral view.

**Figure 5.**
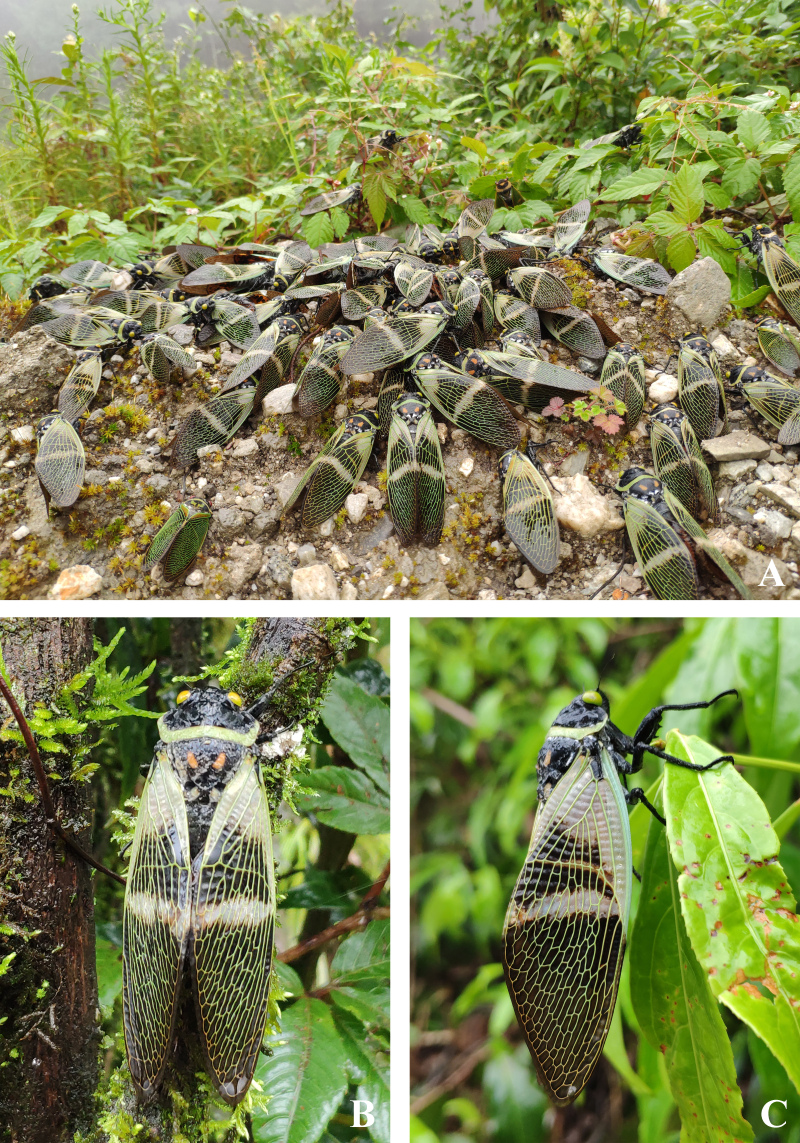
Living *Angamianafloridula* Distant, 1904 at Xiaoheishan Nature Reserve (Yunnan, China). **A** aggregated; **B** dorsal view; **C** lateral view.

**Figure 6.**
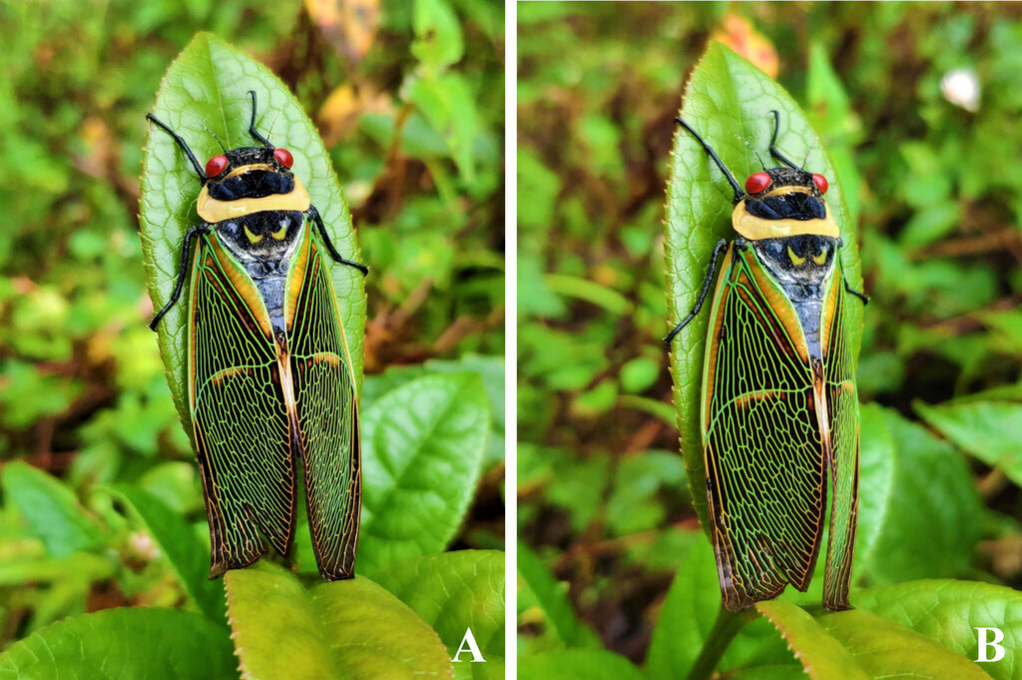
Living *Polyneuracheni* Chou & Yao, 1986 at Xiaoheishan Nature Reserve (Yunnan, China). **A** dorsal view; **B** dorsolateral view.

The new species is bright green in life, with rather reddish compound eyes and with more obvious whitish piles on abdominal tergites (especially dense laterally) and sternites, in line before cruciform elevation and along lateral borders of mesonotum (Fig. 4A–C). As mentioned above, the new species was occurring sympatrically and simultaneously with *Angamianafloridula* Distant, 1904 (Fig. 5) and *Polyneuracheni* Chou & Yao, 1986 (Fig. 6).

## Identification Keys

### Key to species of *Polyneura* Westwood from China

**Table:** 

1	Mesonotum without marks; forewing with apical cells not more than 26	2
–	Mesonotum with yellowish-brown marks; forewing with apical cells about 30	4
2	Forewing with 26 apical cells; pygofer with distal shoulders strongly developed, large, noticeably divergent; uncus wider than long, with uncus lobes much wider and stout, divergent from half length	*P.guoliangi* **sp. n.**
–	Forewing with apical cells not more than 20; pygofer with distal shoulders underdeveloped, small; uncus longer than wide, with uncus lobes relatively short and thin, divergent from basal part	3
3	Pronotum with velutinous setae; forewing with radial cell mostly black-brown, nodal line without light cross band outside; fore trochanter black, without marks	*P.tibetana* Chou & Yao
–	Pronotum with a few setae; forewing with radial cell mostly yellowish-brown, nodal line with short light cross band outside; fore trochanter with three red marks	*P.laevigata* Chou & Yao
4	Abdominal sternite VIII truncated at posterior margin; pronotum with T-shaped mark at anterior margin; nodal line of forewing with long light cross band outside, reaching posterior margin of forewing; ambient vein of hind-wing with pale dark spots inside	*P.xichangensis* Chou & Yao
–	Abdominal sternite VIII rounded at posterior margin; pronotum without T-shaped mark at anterior margin; nodal line of forewing with relatively shorter light cross area outside, not reaching posterior margin of forewing; ambient vein of hind-wing without spots inside	5
5	Cruciform elevation of mesonotum with distinct marks on both sides; abdominal sternite VIII relatively narrow; uncus lobes short, distinctly close together and parallel	*P.parapuncta* Chou & Yao
–	Cruciform elevation of mesonotum without marks on both sides or inconspicuous; abdominal sternite VIII relatively wide; uncus lobes long, distinctly separated	*P.cheni* Chou & Yao

translated and modified from Chou and Yao (1986), Chou et al. (1997)

## Supplementary Material

XML Treatment for Polyneura guoliangi
